# Merging the Multi-Target Effects of Kleeb Bua Daeng, a Thai Traditional Herbal Formula in Unpredictable Chronic Mild Stress-Induced Depression

**DOI:** 10.3390/ph14070659

**Published:** 2021-07-09

**Authors:** Juthamart Maneenet, Orawan Monthakantirat, Supawadee Daodee, Chantana Boonyarat, Yutthana Chotritthirong, Pakakrong Kwankhao, Supaporn Pitiporn, Suresh Awale, Yaowared Chulikhit

**Affiliations:** 1 Graduate School of Pharmaceutical Science, Khon Kaen University, Khon Kaen 40002, Thailand; juthamart_m@kkumail.com (J.M.); yutthana_ch@kkumail.com (Y.C.); 2 Division of Pharmaceutical Chemistry, Faculty of Pharmaceutical Sciences, Khon Kaen University, Khon Kaen 40002, Thailand; oramon@kku.ac.th (O.M.); csupawad@kku.ac.th (S.D.); chaboo@kku.ac.th (C.B.); 3 Department of Pharmacy, Chao Phya Abhaibhubejhr Hospital, Ministry of Public Health, Prachinburi 25000, Thailand; pakakrong2@gmail.com (P.K.); spitiporn@yahoo.com (S.P.); 4 Natural Drug Discovery Laboratory, Institute of Natural Medicine, University of Toyama, 2630 Sugitani, Toyama 930-0194, Japan; suresh@inm.u-toyama.ac.jp

**Keywords:** herbal medicine, KBD formula, *P. nigrum*, *C. asiatica*, *N. nucifera*, UCMS, HPA-axis, depression, neurogenesis, proinflammatory cytokine, MAO inhibition

## Abstract

Major depressive disorder (MDD) is a common and debilitating psychiatric disease characterized by persistent low mood, lack of energy, hypoactivity, anhedonia, decreased libido, and impaired cognitive and social functions. However, the multifactorial etiology of MDD remains largely unknown due the complex interaction between genetics and environment involved. Kleeb Bua Daeng (KBD) is a Thai traditional herbal formula that has been used to promote brain health. It consists of a 1:1:1 ratio of the aerial part of *Centella asiatica*, *Piper nigrum* fruit, and the petals of *Nelumbo nucifera*. According to the pharmacological activities of the individual medicinal plants, KBD has good potential as a treatment for MDD. The present study investigated the antidepressant activity of KBD in an unpredictable chronic mild stress (UCMS) mouse model. Daily administration of KBD to UCMS mice ameliorated both anhedonia, by increasing 2% sucrose intake, and hopeless behavior, by reducing immobility times in the forced swimming test (FST) and tail suspension test (TST) without any effect on locomotor activity. The mechanism of KBD activity was multi-modal. KBD promoted neurogenesis by upregulation of brain-derived neurotrophic factor (BDNF) and cyclic AMP-responsive element binding (CREB) mRNA expression in the frontal cortex and hippocampus. Daily treatment with KBD significantly reversed UCMS-induced HPA axis dysregulation by upregulating the glucocorticoid receptor (GR) while downregulating serum- and glucocorticoid-inducible kinase 1 (SGK1) and FK506 binding protein 5 (FKBP5) mRNA expression. KBD treatment also normalized proinflammatory cytokine expression including tumor necrosis factor-alpha (TNF-α), and interleukin (IL)-1β and IL-6. KBD and its component extracts also exhibited an inhibitory effect in vitro on monoamine oxidase (MAO) A and B. The multiple antidepressant actions of KBD emphasize its potential as an effective, novel treatment for MDD.

## 1. Introduction

Major depressive disorder (MDD) is a common and serious illness presenting a significant socioeconomic burden [1,2]. Depression is a neuropsychiatric syndrome characterized by psychological, behavioral, and physiological symptoms, such as sadness, depressed mood, anhedonia, hopelessness, and suicidal tendencies [3]. The MDD pathogenesis is recognized as being complex, incorporating interactions between genetics, multiple biological systems, and environmental factors. Among these, disruption of monoaminergic function has been the prime hypothesis in MDD pathophysiology, as well as impairment of neurogenesis and neuroplasticity [4]. The brain-derived neurotrophic factor (BDNF) is a neurotrophin related to neuronal growth differentiation and survival that is associated with mood disorders [5,6]. A reduction in BDNF serum levels has been seen in patients diagnosed with MDD, which implies a role for BDNF in the pathology of depression. BDNF expression is regulated by multiple signaling pathways, including cAMP-response element binding (CREB). Long-term treatment with fluoxetine, a selective serotonin reuptake inhibitor, enhances cAMP levels to activate protein kinase A (PKA) and upregulate CREB and BDNF expression in the hippocampus [4]. In addition, there is evidence that the dysregulation of the hypothalamic-pituitary-adrenal (HPA) axis is associated with depression [7,8]. Exposure to stress activates the HPA axis which results in the release of glucocorticoids from the adrenal glands. Glucocorticoids are essential for the maintenance of homeostasis and facilitate the response to physical and emotional stress through the negative feedback regulation of the HPA axis by binding to the glucocorticoid receptor (GR) [9,10]. Under chronic stress conditions, impairment of the GR-mediated negative feedback system of the HPA axis leads to constant HPA axis hyperactivity and chronically high glucocorticoid secretion, which results in the development of depressive disorders. FK506 binding protein 5 (FKBP5) promotes GR stability and reduces GR sensitivity to GC. The upregulation of FKBP5 decreases the ability of GR to bind with glucocorticoid and inhibits the negative feedback loop of the HPA axis [11,12,13]. Serum- and glucocorticoid-inducible kinase 1 (SGK1) has also been implicated in the cellular stress response, as well as in neuronal function. Increased SGK1 function or expression is related to the pathogenic stress hypothesis of MDD [14,15]. On the other hand, clinical and experimental studies reveal that chronic stress and depression are associated with homeostatic imbalances of the immune system, including increased production of pro-inflammatory cytokines such as interleukin (IL)-1, IL-6, tumor necrosis factors (TNFs), and interferons (IFNs) [16,17]. Upregulation of pro-inflammatory cytokines results in neuro-inflammation with associated neuronal structural changes, especially in the hippocampus and prefrontal cortex. The management of MDD has long been based on psychological intervention and pharmacological treatment, but the currently available antidepressants are limited, so interest in alternative and complementary medicines has been growing [18].

Kleeb Bua Daeng (KBD) is a traditional Thai herbal medicine composed of three medicinal plants: black pepper fruits (*Piper nigrum*), the aerial part of Gotu Kola (*Centella asiatica*), and the petal of lotus flowers (*Nelumbo nucifera*), in a 1:1:1 ratio. The KBD formula has been used for more than 70 years by local healers to promote brain health and relieve insomnia. Since June 2013, KBD capsules were officially prescribed in Chao Phya Abhaibhubejr Hospital, which has a reputation for application of Thai traditional medicine and knowledge. The therapeutic effects of KBD could be explained by the medicinal properties of its plant components. There are several reports that have demonstrated antidepressant-like effects of the fruits of *P. nigrum* and the aerial part of *C. asiatica*. A methanolic extract of *P. nigrum* fruits showed antidepressant activity by reducing immobility time in the forced swimming test (FST) in a rat model [19]. Moreover, an ethanolic extract of *C. asiatica* significantly increased the concentration of BDNF and reduced the concentration of TNF-α in the hippocampus of rats in a chronic stress model [20]. In a previous report, we demonstrated that KBD formula improved unpredictable chronic mild stress (UCMS)-induced cognitive dysfunction by a reduction in serum CORT level and brain oxidative damage [21]. KBD also possesses properties associated with the prevention of Alzheimer’s disease including inhibition of acetylcholinesterase, free radical scavenging activities, and reduction of β-amyloid aggregation. Furthermore, KBD also protects against H_2_O_2_-induced neuronal cell death by inhibition of the pro-apoptotic proteins cleaved caspase-9, cleaved caspase-3, p-GSK3β, p-P65, and p-JNK [22]. HPLC analysis of the KBD extract revealed the presence of piperine, madecassoside, asiaticoside, luteolin-7-O-glucoside, rutin, kaempferol-3-glucoside, quercetin, kaempferol, and ferulic acid as major constituents [21]. The chemical constituents of KBD extract that are relevant to its pharmacological activities.

Due to the multiple targets in the pathological cascade of MDD, classical treatments that modulate only one target of action may be inadequate [23]. Thus, alternative therapeutic approaches targeting different biological pathways are being explored to improve treatment outcomes. Herbal medicines are a natural, rich, and diverse source of chemicals that could target multiple sites in the pathology of depression. Therefore, the KBD formula, which consists of three herbal medicines, could provide additional advantages for MDD treatment. In addition, there has been no previous study investigating KBD as a therapeutic for depression. In order to ascertain the neuropharmacological activity of KBD, the same batch of KBD capsule was used to evaluated their effect on UCMS-induced depressive symptoms in mice and assessed changes in expression of BDNF, CREB, GR, SGK1, FKBP5, IL-1β, IL-6, and TNF-α in the frontal cortex and hippocampus regions. In vitro MAO-A and MAO-B inhibitory effects of KBD extract were also performed.

## 2. Results

### 2.1. Effects of Kleeb Bua Daeng Formula on UCMS-Induced Anhedonia in Mice Using the Sucrose Preference Test

**Figure 1:**
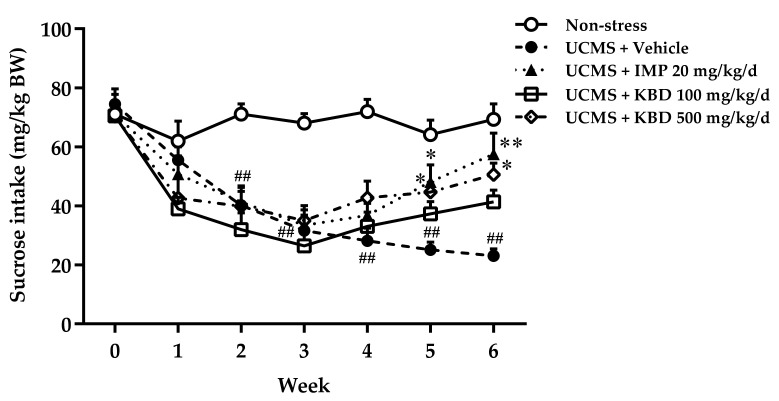
Effect of daily imipramine or KBD formula treatments on UCMS-induced anhedonia. Reductions in the amount of 2% sucrose solution taken indicate anhedonia. Each data point represents the mean ± S.E.M. (*n* = 10 in each group). ^##^ *p* < 0.001 vs. vehicle-treated non-stress group. * *p* < 0.05 and ** *p* < 0.001 vs. vehicle-treated UCMS group.

The sucrose preference test was performed to examine whether the UCMS procedure induced anhedonia in mice in this study. As shown in Figure 1, the mice in the vehicle-treated UCMS group consumed significantly less 2% sucrose solution than the vehicle-treated non-stress group from 2 weeks after starting the UCMS procedure, indicating the onset of anhedonia. Daily administration of either 500 mg/kg KBD formula or 20 mg/kg imipramine to UCMS mice significantly increased their 2% sucrose solution consumption in weeks 5 and 6 when compared with mice in the vehicle-treated UCMS group, indicating a reduction in anhedonic behavior.

### 2.2. Effects of Kleeb Bua Daeng Formula on UCMS-Induced Hopeless Behavior in Mice Using the Forced Swimming and Tail Suspension Tests

**Figure 2:**
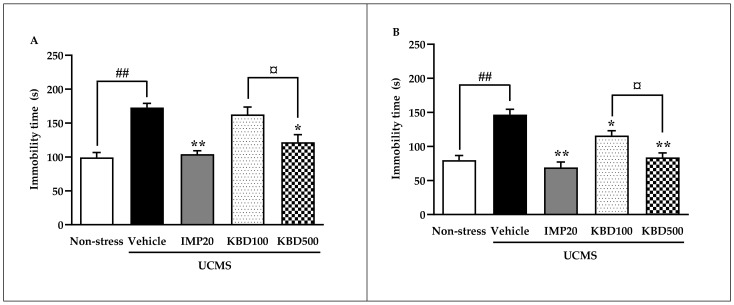
Effect of daily imipramine and KBD formula treatments on UCMS-induced hopeless behavior in the forced swimming test (**A**) and tail suspension test (**B**). Increased immobility times indicate hopeless behavior. Each data column represents the mean ± S.E.M. (*n* = 10–12 in each group). ^##^ *p* < 0.001 vs. vehicle-treated non-stress group. * *p* < 0.05 and ** *p* < 0.001 vs. vehicle-treated UCMS group. ^¤^ *p* < 0.05 compared between doses of the KBD formula.

The forced swimming test (FST) and tail suspension test (TST) were performed in this study to assess hopeless behavior. As shown in Figure 2, immobility times were significantly increased in the vehicle-treated UCMS group when compared with vehicle-treated non-stress group in both the FST and TST, indicating UCMS-induced hopeless behavior. Daily treatment with 20 mg/kg imipramine significantly decreased immobility times in both the FST and TST compared to the vehicle-treated UCMS group. KBD treatment significantly decreased immobility times in both the FST and TST compared to the vehicle-treated UCMS group only at dose 500 mg/kg, indicating that KBD reduced UCMS-induced hopeless behavior in a dose-dependent manner.

### 2.3. Effects of UCMS and Kleeb Bua Daeng Formula on the Locomotor Activity of Mice Using the Y-Maze Test

**Figure 3:**
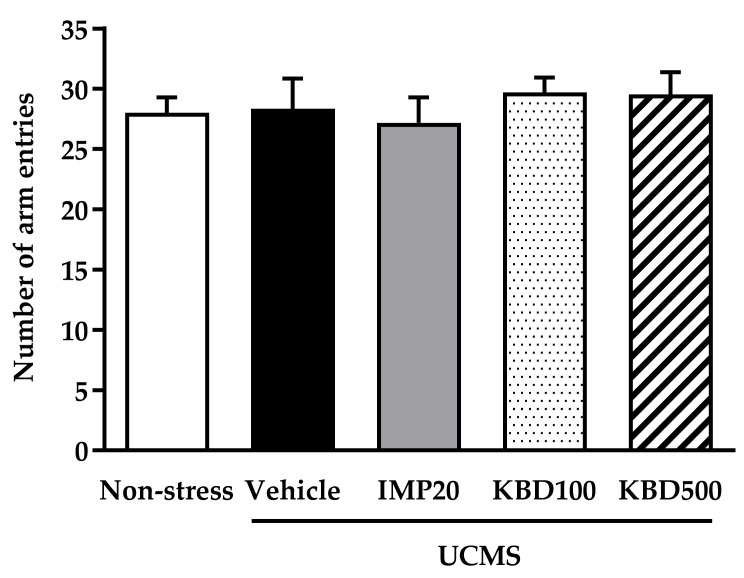
Effect of the UCMS procedure and daily treatment with imipramine and KBD formula on locomotor activity in the Y-maze test. The total number of arm entries was determined in each group. Reductions in the number of arm entries are indicative of depression. Each data column represents the mean ± S.E.M. (*n* = 10 in each group).

To evaluate whether the UCMS, KBD, or IMP treatments affected mouse locomotor activity, the Y-maze test was performed. The total number of arm entries in the Y-maze was determined in each group. Reductions in the number of arm entries are indicative of depression. The results showed that locomotor activity was not significantly affected by any treatments (Figure 3).

### 2.4. Effect of Kleeb Bua Daeng Formula on UCMS-Induced Changes to the Frontal Cortex and Hippocampus Brain Regions of Mice

**Figure 4:**
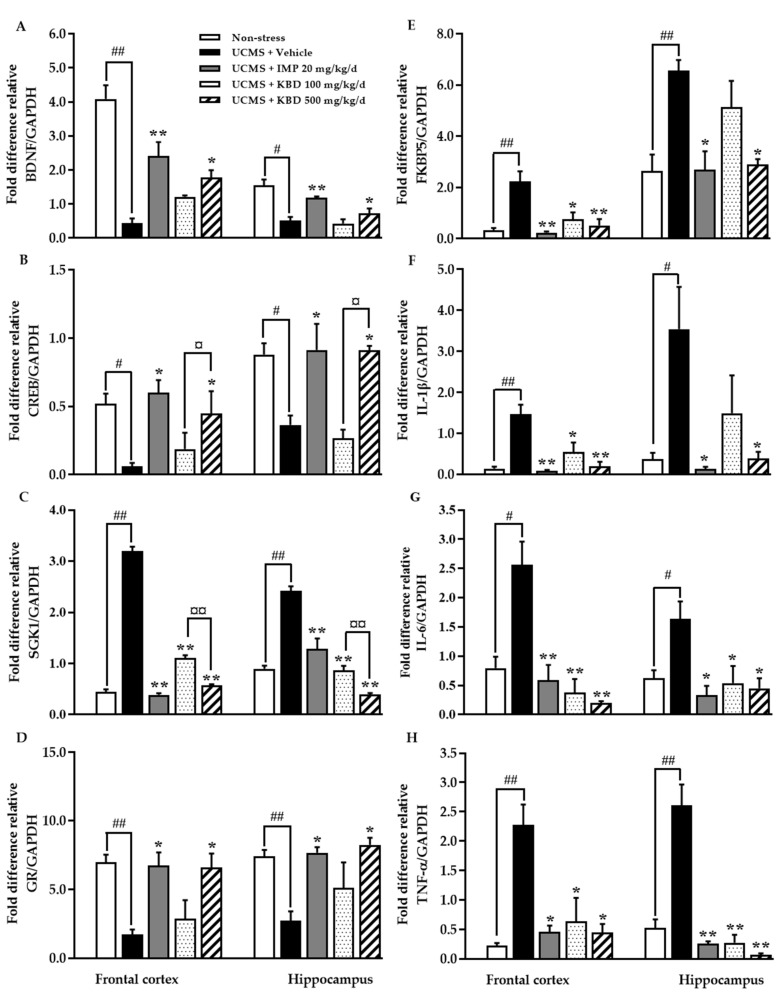
Effect of daily imipramine and KBD formula treatments on UCMS-induced changes in depressive-related mRNA expression in the frontal cortex and hippocampus regions of mouse brain ((**A**) = BDNF; (**B**) = CREB; (**C**) = SGK1; (**D**) = GR; (**E**) = FKBP5; (**F**) = IL-1β; (**G**) = IL-6, and (**H**) = TNF-α). Each data column represents the mean ± S.E.M. (*n* = 5 in each group). ^#^ *p* < 0.05 and ^##^ *p* < 0.001 vs. vehicle-treated non-stress group. * *p* < 0.05 and ** *p* < 0.001 vs. vehicle-treated UCMS group. **^¤^** *p* < 0.05 and **^¤¤^** *p* < 0.001 compared between doses of the KBD formula.

Quantitative real time PCR (QPCR) analysis was performed to assess expression of genes encoding BDNF, CREB, GR, SGK1, FKBP5, IL-1β, IL-6, and TNF-α in the frontal cortex and hippocampus regions of mouse brains. As shown in Figure 4, mRNA expression of BDNF, CREB, and GR was significantly decreased and mRNA expression of SGK1, FKBP5, IL-1β, IL-6, and TNF-α was significantly increased in vehicle-treated UCMS mice in both the frontal cortex and hippocampus when compared with vehicle-treated non-stress mice. Daily treatment with either 500 mg/kg KBD formula or 20 mg/kg imipramine restored expression levels; significantly increasing BDNF, CREB, and GR mRNA expression and significantly decreasing SGK1, FKBP5, IL-1β, IL-6, and TNF-α mRNA expression in both brain regions.

### 2.5. Effect of Kleeb Bua Daeng and Its Component Extracts on Monoamine Oxidase (MAO)-A and MAO-B Activity

The MAO-A and MAO-B inhibitory effect of KBD extracts and its components *P. nigrum*, *C. asiatica,* and *N. nucifera* were performed and their MAO-A and MAO-B inhibitory IC_50_ values were calculated and reported as shown in Table 1. Among all extracts, *C. asiatica* was found as the most active MAO-A inhibitor with IC_50_ value of 127 ug/mL and KBD was found as the most active MAO-B inhibitor with IC_50_ value of 110 ug/mL. The IC_50_ values were converted to the corresponding enzyme-inhibitor dissociation constants (*Ki* values) using the Cheng–Prusoff equation [6]. The *Ki* values allowed the calculation of the selective index for MAO-A/B, and the results are shown in Table 1. The selectivity index for MAO-A and MAO-B isoforms indicates that *N. nucifera* was partially selective for the MAO-A isoform, which is specific for antidepressant activity.

## 3. Discussion

**Figure 5:**
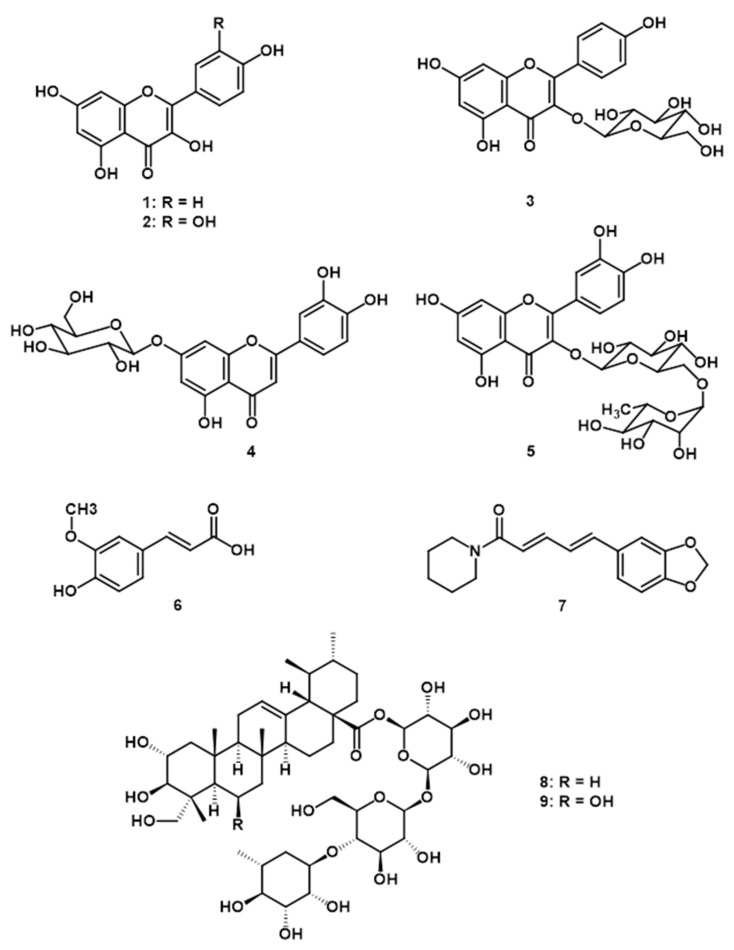
Structures of kaempferol (**1**), quercetin (**2**), kaempferol-3-glucoside (**3**), luteolin-7-*O*-glucoside (**4**), rutin (**5**), ferulic acid (**6**), piperine (**7**), asiaticoside (**8**), and madecassoside (**9**) [21].

KBD, a Thai traditional herbal formula, consists of three herbal plants, i.e., *Piper nigrum*, *Centella asiatica*, and *Nelumbo nucifera*. In a previous study we demonstrated that the KBD formula exerted neuroprotective activity in an Alzheimer’s mouse model by enhancing learning and memory performance [21,22]. In addition, we recently investigated the composition of the KBD formula by HPLC fingerprint method and we found kaempferol (1), quercetin (2), kaempferol-3-glucoside (3), luteolin-7-*O*-glucoside (4), rutin (5), ferulic acid (6), piperine (7), asiaticoside (8), and madecassoside (9) (Figure 5) [21]. According to its ethno-medical uses and chemical constituents in each medicinal plant, these suggest that KBD may have potential for the treatment of neuropsychiatric symptoms. Hence, this current investigation used a UCMS animal model of depression to evaluate the antidepressant-like effects of KBD formula and elucidate its mechanisms of action. UCMS induced anhedonia and hopeless behavior in mice, and changed expression levels of the depression-related genes BDNF, CREB, GR, SGK1, FKBP5, IL-1β, IL-6, and TNF-α in the frontal cortex and hippocampus. Daily administration of the KBD formula ameliorated these depressive behaviors by reversing the effects of UCMS-induced reduction of neurogenesis, HPA axis dysregulation, and neuro-inflammation in the hippocampus and frontal cortex.

Although depression is highly prevalent, its etiology remains unclear and the presently available antidepressant medications are only moderately effective [23,24]. Animal models can provide insights into the pathogenic and neurobiological mechanisms of depression and allow a better understanding of the inner working of the brain. There are several animal models of depression such as the UCMS procedure, subcutaneous administration of corticosteroids, social isolation, and olfactory bulbectomy [25]. The UCMS model is one of the most well-used models and has been used as an animal model of depression for several years [26]. Chronic stress induces depression by presenting long term unpredictable and uncontrollable stress stimuli, which is similar to conditions associated with depressed patients [27]. Several neurobiological abnormalities and symptoms of the UCMS-induced animals are similar to those manifested in MDD patients such as high serum CORT level, hippocampus atrophy, hopelessness and anhedonia behaviors [6,11]

The first experiments were designed to evaluate the effects of the KBD formula on UCMS-induced anhedonia using the sucrose preference test and hopeless behavior using FST and TST. Anhedonia (the loss of pleasure or lack of reactivity to pleasurable stimuli) and hopelessness are core symptoms of depressed patients and are the most promising endophenotypes of depression [28,29,30]. Accumulating evidence has demonstrated that the UCMS procedure induces anhedonia and hopeless behavior, which can be recovered by antidepressant drugs [5]. Daily administration of the KBD formula ameliorated the UCMS-induced reduction in sucrose solution intake according to the sucrose preference test and reversed hopeless behavior as effectively as imipramine, the reference antidepressant drug. In order to exclude the false positive of mice movement caused by drug-induced hyperlocomotion in the hopeless behavioral tests, a Y-maze test was performed to investigate locomotor activity. We found that neither imipramine nor KBD formula altered the locomotor activity of UCMS mice indicating that the KBD formula can ameliorate the anhedonia and hopeless behavior caused by UCMS.

Due to the multifaceted pathogenesis of depression, this study aimed to clarify whether the KBD formula ameliorated the effect of UCMS-induced changes in expression levels of the depression-related genes BDNF, CREB, GR, SGK1, FKBP5, IL-1β, IL-6, and TNF-α. The neurotrophic hypothesis postulates that neuronal plasticity is a key factor in development of depression and in the clinical response to antidepressants [31]. BDNF, a member of the neurotrophin family, is a growth factor important for cell survival, neurogenesis, synaptogenesis, and neuroplasticity, with inadequate levels associated with cell atrophy in the hippocampus and prefrontal areas of the brain that have, in turn, been implicated in vulnerability to depression [32,33]. BNDF expression is regulated by multiple signaling pathways, including CREB which is one of the best studied transcription factors implicated in depression and antidepressant-like responses [34]. The role of CREB-BDNF in depression is via tyrosine receptor kinase B (TrkB), a member of the tyrosine kinase family, that can specifically bind to BDNF with a high affinity. Intracellular tyrosine kinase activity is activated by BDNF binding to TrkB, leading to the activation of the mitogen-activated protein kinase (MAPK) pathway, the phospholipase C-gamma (PLC-γ) pathway, the phosphatidylinositol 3-kinase (PI3K) pathway, and other kinase signaling pathways [35]. The accumulated evidence suggests that CREB-BDNF signaling is critical in numerous neuronal biological processes, including cell survival, synaptic structure, and synaptic plasticity [36]. Exposure to stress reduces the hippocampal expression of CREB-BDNF mRNA via the MAPK, PLC-γ and PI3K signal transduction pathways, [35]. It has been suggested that antidepressants upregulate cellular PKA activity and increase the translocation of PKA to the nucleus by recruiting CREB to upregulate neurogenesis and neuronal plasticity, which underlies their antidepressant activity [37,38]. Our results demonstrated that BDNF and CREB mRNA expression levels in the frontal cortex and hippocampus were reduced in UCMS mice when compared with non-stress mice and UCMS mice treated with KBD formula or imipramine showed significantly increased expression of BDNF and CREB mRNA in both brain regions (see Figure 4). In accordance with our results, Xu and colleagues found that chronic stress induced downregulation of BDNF protein levels and decreased the ratio of phosphorylated CREB (pCREB) to CREB levels (pCREB/CREB) in rat frontal cortex and hippocampus [39]. Thus, the UCMS model induces a reduction in CREB/BDNF expression levels, which decreases cell survival, synaptic structure, and synaptic plasticity leading to downregulation of cell proliferation and neurogenesis [40].

The HPA axis is the major system involved in the stress response. The activation of the HPA axis results in the release of glucocorticoid from the adrenal gland which promotes physiologic stress by preparing the fight and flight response. These stress responses are also critical for terminating the response via negative feedback on several levels of the HPA axis via activation of the glucocorticoid receptor (GR). Under pathological conditions, impairment of the GR-mediated negative feedback system leads to constant HPA axis hyperactivity and chronically high GC levels, resulting in the development of depressive disorders. The normalization of GR function is a key to antidepressant action [41]. GR is kept in an inactive state in the cytoplasm, only translocating from the cytoplasm to the nucleus to execute its functions when it binds to glucocorticoid. Impaired signaling via corticosteroid-activated GR leads to impaired negative feedback regulation and partial glucocorticoid resistance appears to be one of the most robust biological abnormalities observed in mood disorders [42]. FKBP5, an Hsp90 co-chaperone is a negative modulator of GR activity that promotes GR stability and reduces GR sensitivity to glucocorticoid. When FKBP5 is bound to GRs, the GR has a lower binding affinity for glucocorticoid and is retained in the cytoplasm resulting in the inhibition of the negative feedback loop of the HPA axis. It has been found that both chronic CORT/dexamethasone exposure and UCMS can increase either the expression of FKBP5 mRNA, protein, or both, in rodent brain, particularly in the hippocampus and the frontal cortex [43,44,45,46]. Antidepressants can reverse these increases in the mRNA and protein expression of FKBP5 in either the hippocampus, prefrontal cortex, or both, of CMS-exposed rats [43]. In line with previous research, our findings show that UCMS exposure significantly increased expression of FKBP5. KBD and imipramine reversed the UCMS-induced increase in FKBP5 mRNA expression in the hippocampus and frontal cortex, which provides evidence for the specific targets of KBD in the treatment of depression. In addition, our prior research has also demonstrated that chronic exposure to UCMS significantly increases the serum CORT level and KBD treatment restores serum CORT levels to normal [21]. In this context, we first assessed the effect of KBD on GR mRNA expression in the hippocampus and frontal cortex of UCMS mice. Consistent with previous findings, we demonstrated that UCMS exposure downregulated GR mRNA in the hippocampus and frontal cortex. Treatment with KBD significantly restored GR function to levels similar to imipramine. SGK1, another GR target gene, is a serine/threonine kinase belonging to the AGK kinase family. Although SGK1 was initially described for its role in regulating sodium transport in renal collecting duct cells [15], recent studies have provided evidence for a role of SGK1 in stress and glucocorticoid actions on the brain. Compelling data suggests that SGK1 is involved in the GC-induced reduction in the proliferation and differentiation of human hippocampal progenitor cells and that increased SGK1 expression or function is related to the pathogenic stress hypothesis of major depression [42]. SGK1 mRNA expression has been found to be significantly increased (positive correlation with FKBP mRNA expression) in the peripheral blood of drug-free depressive patients, as well as in the hippocampus of rats subjected to chronic stress [15]. In the present study, UCMS exposure consistently and significantly increased the mRNA expression of SGK1 in both the hippocampus and frontal cortex. The administration of KBD and imipramine markedly suppressed the increase in SGK1 mRNA expression in a dose-dependent manner. Taken together, these findings reveal the potential molecular mechanisms by which KBD is involved in FKBP5- and SGK1-mediated GR activation and normalization of glucocorticoid rhythms in the HPA axis (see Figure 4).

Similarly, it is of interest that the KBD formula reversed the UCMS-induced increased mRNA expression levels of IL-1β, IL-6, and TNF-α in both the frontal cortex and hippocampus (see Figure 4). The immune system is implicated in the pathogenesis of depression through proinflammatory cytokines, which are behavioral, neuroendocrine, and neurochemical mediators of depression [46]. Chronic stress leads to perturbations in the immune system and elevates production of proinflammatory cytokines [47]. Increased TNF-α, one of the most important proinflammatory cytokines families, has been reported in clinical depression [48,49]. More significantly, some proinflammatory cytokines are reduced by some antidepressant drugs [50]. For example, sleep deprivation has been related to higher levels of IL-6, and the dysregulation of sleep is common in patients with depression [50]. As well as IL-6, IL-1β is one factor with notable implication in the pathophysiology of depression. IL-1β levels have been reported to be high in depressed elderly patients [51,52,53]. This data supports a role for chronic stress induced proinflammatory cytokines in depressive illness. Yang and colleagues investigated UCMS-induced increases in proinflammatory cytokines in the rat brain. They found that stressed rats displayed more IL-1β positive cells in the hippocampus while many more IL-6 positive cells were shown in caudate putamen and ventromedial hypothalamus. Interestingly, TNF-α positive cells were found to be more common in the prefrontal cortex and hippocampus [50]. KBD formula significantly decreased these proinflammatory cytokines in our UCMS-induced mice suggesting that KBD might also ameliorate the levels of inflammatory cytokines in depressed patients.

The MAO inhibitor hypothesis suggests that MAO plays an important role in depressive disorder. MAO has two isozymes, MAO-A which metabolizes serotonin and noradrenaline, and MAO-B which prefers phenylethylamine, methylhistamine, and tryptamine. Whereas dopamine and tyramine are metabolized by both isoenzymes [54]. Decreased levels of monoamine transmitters is a related cause of depression [55,56]. Thus, KBD and its components’ in vitro monoamine oxidase inhibitory activities were analyzed. The selectivity index for MAO-A and MAO-B isoforms indicates that and *N. nucifera* was partially selective for the MAO-A isoform, which is specific for antidepressant activity. These results suggest that one possible mechanism of the KBD extract antidepressant activity is particularly involved in MAO inhibitory effect (see Table 1).

A recent report analyzed the phytochemical composition of KBD extract using high performance liquid chromatography (HPLC). The HPLC fingerprint revealed the presence of piperine, madecassoside, asiaticoside, quercetin, kaempferol, kaempferol-3-glucoside, rutin, luteolin-7-*O*-glucoside, and ferulic acid. The amount of each compound was also quantitatively analysed by HPLC and we found that the major components in this KBD formula were madecassoside (179 mg/g extract), asiaticoside (57 mg/g extract), and piperine (10 mg/g extract), respectively [21]. Accumulation evidences demonstrate that the chemical constituents of KBD extract which are relevant to antidepressant activity are madecassoside, asiaticoside, piperine, quercetin, kaempferol, kaempferol-3-glucoside, rutin, luteolin-7-*O*-glucoside, and ferulic acid [21,57,58,59,60,61,62,63]. Asiaticoside, one of the triterpenoid compounds isolated from *C. asiatica*, has been demonstrated to increase sucrose consumption and reduced immobility time in FST and TST, and downregulate IL-1β, IL-6, and TNF-α in the hippocampus [57]. In addition, madecassoside also produces antidepressant effects through MAO inhibition in rat brain [58]. Piperine, the major compound isolated from *P. nigrum*, has been investigated for antidepressant-like effects in a corticosterone administration model. The results showed that piperine reversed the effect of corticosterone treatment via increased BDNF protein and mRNA levels in the hippocampus [59]. A recently report studying the antidepressant-like effects of luteolin showed that it elevated the monoamine neurotransmitters levels in the synaptic cleft and upregulated BDNF expression in hippocampus [60]. Kaempferol and quercetin, two flavonoids commonly found in medicinal plants exhibited antidepressant activity by reducing the immobility time in FST and TST in stressed mice compared with unstressed mice [61]. Rutin alleviated chronic unpredictable stress-induced depressive behavioral changes and damage to the hippocampus in mice [62]. Ferulic acid exerted an antidepressant-like effect in a TST mouse model [63] and inhibited MAO-A activity in the frontal cortex and hippocampus [64]. This evidence supports our findings of the antidepressant effects of the KBD formula in this investigation.

## 4. Materials and Methods

### 4.1. Preparation of Kleeb Bua Daeng Extract

The Thai traditional formula, Kleeb Bua Daeng (KBD), was provided Chao Phya Abhaibhubejhr Hospital, Prachinburi Province, Thailand. The KBD formula consists of three medicinal plants: *N. nucifera* petals, the aerial part of *C. asiatica*, and *P. nigrum* fruits. The plants were identified by Benjawan Leenin, a chief at the Traditional Knowledge Center, Chao Phya Abhaibhubejhr Hospital Foundation. The relative herbarium voucher specimens were deposited in the Chao Phya Abhaibhubejhr Hospital museum with the following voucher numbers: ABH15, ABH17, and ABH18, respectively. Dried powdered KBD formula and its components (300 g in each sample) were macerated with 95% ethanol at room temperature (3 × 1.5 L, three days/cycle). The extracts were combined and concentrated under reduced pressure at 50 °C. The extracts were freeze dried and kept at ‒20 °C throughout the experiment. The HPLC analysis of the KBD extract was reported in our previous study [21]. The results revealed the presence of piperine, madecassoside, asiaticoside, luteolin-7-*O*-glucoside, rutin, kaempferol-3-glucoside, quercetin, kaempferol, and ferulic acid as major constituents in the KBD formula [21].

### 4.2. Animals and Ethics

Five-week-old male ICR mice (20–30 g) were obtained from Nomura Siam International. Mice were housed on wood chip bedding in stainless steel cages with free access to food and water. Housing was thermostatically maintained at 22 ± 2 °C with constant humidity (45 ± 2%) and a 12 h light-dark cycle (light on: 06:00–18:00). The experimental protocols were in accordance with the Guiding Principles for the Care and Use of Animal (NIH Publications #8–23, revised in 2011) and were approved by the Animal Ethics Committee for Use and Care of Khon Kaen University, Khon Kaen, Thailand (approval No. IACUC-KKU-36/61, 21 Jun 2018).

### 4.3. Unpredictable Chronic Mild Stress

**Figure 6:**
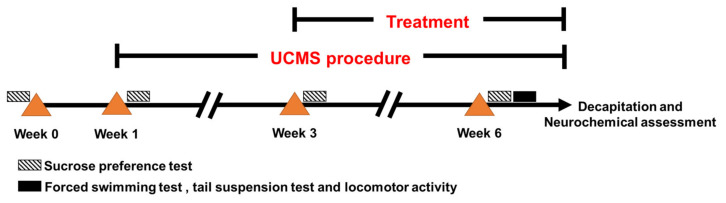
Schematic of experiment protocol. Mice were divided into five groups; a non-stress group administered 0.5% sodium carboxymethyl cellulose (SCMC) daily for 6 weeks (non-stress), and four UCMS groups. The UCMS groups were exposed to various stressors for 6 weeks. The UCMS mice were divided into four groups which were administered 0.5% SCMC (UCMS + vehicle), 20 mg/kg i.p. imipramine (UCMS + IMP20), 100 mg/kg p.o. KBD (UCMS + KBD100), or 500 mg/kg p.o. KBD (UCMS + KBD500) daily from week 3. The sucrose preference test was performed every week during the UCMS procedure. The forced swimming test (FST), tail suspension test (TST), and assessment of locomotor activity were conducted after week 6. After finishing the behavioral tests, all animals were decapitated, and their brains were collected for neurochemical assessment.

This investigation was conducted according to the experiment protocols described in Figure 6. Unpredictable chronic mild stress (UCMS) involves the exposure of mice to a variety of mild unpredictable stressors in a random order over several weeks. In the UCMS procedure, mice were divided into 5 groups; a non-stress group and 4 groups subjected to UCMS for 6 weeks. The UCMS schedule consisted of a variety of stressors including one 18-h period of food and water deprivation, two 12-h periods of cage tilting at 45°, two 1-h periods of restricted access to food micro pellets (1 h), one 21-h period of a wet cage (200 mL of water in 100 g of wood chip bedding), two 36-h periods of continuous light exposure, two periods of exposure to intermittent sound (3 and 5 h), two 2-h periods of paired caging, and two 3-h periods with an empty water bottle.

### 4.4. Drug Administration

This preparation of drug administration, 0.5 g sodium carboxymethyl cellulose (SCMC), was dissolved with distilled water (100 mL). KBD powder (100 and 500 mg) was dissolved with 0.5% SCMC (10 mL) while imipramine (20 mg) was dissolved with 0.9% normal saline (1 mL). Mice were randomly divided into 5 groups (*n* = 12 each). The first group was the non-stress group and received vehicle (0.5% SCMC/day, p.o.). Furthermore, five UCMS groups received vehicle daily (0.5% SCMC/day, p.o.), imipramine (20 mg/kg/day, i.p.), low dose of KBD (100 mg/kg/day, p.o.) and high dose of KBD (500 mg/kg/day, p.o.). All treatments were administered daily for 3 weeks after day 21 at 8:00 a.m. except on behavioral testing day where mice were administered treatments 1 h before testing.

### 4.5. Sucrose Preference Test

Anhedonia behavior was assessed by the sucrose preference test. The sucrose preference test was conducted before the start of the UCMS procedure and once a week during the UCMS procedure. Eighteen-hours after food and water deprivation, mice were individually placed in cages and received 2% sucrose solution for 1 h. The amount of sucrose solution consumed during 1-h was recorded.

### 4.6. Forced Swimming Test

The forced swimming test (FST) is one of the most commonly used animal models for assessing antidepressant-like behavior [65]. FST involves the scoring of active (swimming and climbing) or passive (immobile) behavior when mice are forced to swim in a cylinder from which there is no escape [66]. The FST was conducted as previously described [63]. Briefly, mice were placed individually in transparent glass cylinders (12 cm in diameter, height 25 cm) filled to a height of 10 cm with water at 25 °C. The FST was divided into two sessions; pre-test and test sessions. In the pre-test session, mice were forced to swim for 15 min, 24 h before the test session. During the test session, mice were administered the drug 1 h before the test and placed in the same conditions. The immobility time was recorded for 5 min.

### 4.7. Tail Suspension Test

In the tail suspension test (TST, mice are suspended upside down, which leads to characteristic behavioral immobility which resembles human depression [67]. The TST was carried out as previous described [68]. Briefly, mice were individually suspended in a suspension test box by adhesive tape placed approximately 1 cm from the tip of the tail, 60 cm above the surface of table. Immobility duration was recorded for the last 4 min during a 6 min period. Mice were considered immobile when they hanged passively and completely motionless [66].

### 4.8. Locomotor Activity

The Y-maze task was applied to determine locomotor activity. Mice were placed in a Y-maze and the total number of arm entries were recorded over an 8-min period [69].

### 4.9. Quantitative Real Time PCR

Mouse brain-derived neurotrophic factor (BDNF), cyclic AMP-responsive element binding (CREB), glucocorticoid receptor (GR), serum- and glucocorticoid-inducible kinase 1 (SGK1), FK506 binding protein 5 (FKBP5), interleukin (IL)-1β, IL-6, and tumor necrosis factor-alpha (TNF-α) expression levels from frontal cortex and hippocampus brain were analyzed by quantitative real-time polymerase chain reaction (Q-PCR). Total RNA from the frontal cortex and hippocampus were extracted with TRIzol reagent (Thermo Fisher Scientific Inc., San Jose, CA, USA), according to instructions. The reaction mixture consisted of 10 µM of primer pairs (0.5 µL), RNase free water (4 µL), SsoAdvancedTM Universal SYBR Green Supermix (Biorad, Hercules, CA, USA) (10 µL) and 10 ng/mL of cDNA (5 µL). The following primers were synthesized by Macrogen (South Korea): GADPH, 5′-ACC ACA GTC CAT GCC ATC AC-3′ (forward) and 5′-TCC ACC ACC CTG TTG CTG TA-3′ (reverse); BNDF, 5′-GAC AAG GCA ACT TGG CCT AC-3′ (forward) and 5′-CCT GTC ACA CAC GCT CAG CTC-3′ (reverse); CREB, 5′-TAC CCA GGG AGG AGG AAT AC-3′ (forward) and 5′-GAG GCT GCT TGA ACA ACA AC-3′ (reverse); GR, 5′- CAC TAA TCC TCT CCA TCC TAC-3′ (forward) and 5′- AAT GTC TGC TGC CTT CTG-3′ (reverse); SGK1, 5′-GGG TGC CAA GGA TGA CTT TA-3′ (forward) and 5′-CTC GGT AAA CTC GGG ATA GA-3′ (reverse); FKBP5, 5′- GAA CCC AAT GCT GAG CTT ATG-3′ (forward) and 5′- ATG TAC TTG CCT CCC TTG AAG -3′ (reverse); IL-1β, 5′-GAC AGC AA GTG ATA GGC C-3′ (forward) and 5′-CGT CGG CAA TGT ATG TGT TGG-3′ (reverse); IL-6, 5′-CTT CCA TCC AGT TGC CTT CTT-3′ (forward) and 5′-AAT TAA GCC TCC GAC TTG TGA AG-3′ (reverse); TNF-α, 5′-GCC TCT TCT CAT TCC TGC TTG-3′ (forward) and 5′-CTG ATG AGA GGG AGG CCA TT-3′ (reverse). In this study, glyceraldehyde 3-phosphate dehydrogenase (GAPDH) was used as a housekeeping gene. The results were calculated and were expressed as fold-differences.

### 4.10. Human Monoamine Oxidase A and B Inhibitory Activity Assay

The monoamine oxidase (MAO) inhibitory properties of KBD extract and each component in KBD formula (*P. nigrum*, *C. asiatica*, and *N. nucifera*) were evaluated using human MAO–A and –B as enzyme sources and kynuramine as a substrate. Clorgyline and deprenyl were used as standard references for selective MAO–A and –B inhibitors, respectively. Furthermore, 100 mg of test sample was dissolved in 400 µL DMSO to prepare 250 mg/mL as a stock solution. The reaction mixtures contained kynuramine (45 and 30 µM for MAO–A and –B, respectively), test samples (containing 4% DMSO), potassium phosphate buffer (pH 7.4), and MAO inhibitors (0.075 mg/mL). Test samples were serial diluted as a working solution. Each concentration (20 µL) was mixed with kynuramine (9 and 6 µL for MAO–A and –B, respectively) and potassium phosphate buffer (469.5 and 472.5 µL for MAO–A and –B, respectively). MAO–A or –B enzymes (1.5 µL) were added to the reaction and the mixture was incubated at 37 °C for 20 min. The reaction was subsequently terminated by addition of 400 µL NaOH (2 N) and 1000 µL of distilled water. Fluorescence of 4-hydroxyquinoline was measured at excitation and emission wavelengths of 310 and 400 nm, respectively. The IC_50_ values were calculated and converted to the corresponding *Ki* values according to the Cheng–Prusoff equation [6]. The *Ki* values allowed the calculation of the MAO-A/B selectivity ratios (Si = *Ki* (MAO-B)/*Ki* (MAO-B)).

### 4.11. Statistical Analysis

The results are expressed as mean ± S.E.M. T-test was performed for comparison between non-stress and UCMS groups. The analysis was performed by one-way analysis of variance (ANOVA) followed by the Tukey test for multiple comparisons among different groups. Differences with *p* < 0.05 were considered significant.

## 5. Conclusions

In the present study, we demonstrated the experimental evidence merging the multi-faceted beneficial effects of KBD formula. This may be a plus in the fight against stress-induced depression since a multi-target intervention appears more promising compared with precision medicine. The KBD formula normalized UMCS-induced anhedonia and hopeless behaviors in mice by restoring expression of the depression-related genes BDNF, CREB, GR, SGK1, FKBP5, IL-1β, IL-6, and TNF-α in the frontal cortex and hippocampus brain regions. Daily treatment with the KBD formula demonstrated similar activity as imipramine, the reference antidepressant drug. KBD and its component extracts also exhibited selective inhibition of MAO-B and partial selective inhibition of MAO-A. These results clarify the antidepressant mechanisms of the KBD formula and illustrate the potential of this traditional medicine with depression-regulation properties.

## Figures and Tables

**Table 1:** Monoamine oxidase inhibitory activities of KBD formula and its component extracts.

Extract	IC_50_ (µg/mL)		*Ki* (µg/mL) ^3^		Si ^4^	
	MAO-A	MAO-B	MAO-A	MAO-B	MAO-A	MAO-B
KBD	342.4 ± 0.849	110.2 ± 0.546	90.223	47.425	1.903	0.526
*P. nigrum*	396.7 ± 0.058	211.9 ± 0.055	104.531	97.274	1.145	0.873
*C. asiatica*	127.0 ± 0.065	141.3 ± 0.106	33.465	60.864	0.550	1.819
*N. nucifera*	141.9 ± 0.065	412.3 ± 0.220	37.391	177.594	0.211	4.750
Clorgyline ^1^	0.0015 ± 0.00001 µM	2.99 ± 0.162 µM	0.0004	1.288	0.00031	3213.46
Deprenyl ^2^	687.6 ± 4.879 µM	0.291 ± 0.044 µM	181.200	0.125	1445.58	0.0007

^1^ Clorgyline is a selective MAO-A inhibitor. ^2^ Deprenyl is a selective MAO-B inhibitor. ^3^ Enzyme inhibitor dissociation constant. ^4^ Selectivity index.

## Data Availability

Data is contained within the article.
